# Fragile X Syndrome: From Molecular Aspect to Clinical Treatment

**DOI:** 10.3390/ijms23041935

**Published:** 2022-02-09

**Authors:** Dragana D. Protic, Ramkumar Aishworiya, Maria Jimena Salcedo-Arellano, Si Jie Tang, Jelena Milisavljevic, Filip Mitrovic, Randi J. Hagerman, Dejan B. Budimirovic

**Affiliations:** 1 Department of Pharmacology, Clinical Pharmacology and Toxicology, Faculty of Medicine, University of Belgrade, 11129 Belgrade, Serbia; 2 Medical Investigation of Neurodevelopmental Disorders (MIND) Institute UCDH, University of California Davis, 2825 50th Street, Sacramento, CA 95817, USA; aramkumar@ucdavis.edu (R.A.); mjsalcedo@ucdavis.edu (M.J.S.-A.); sijtang@ucdavis.edu (S.J.T.); rjhagerman@ucdavis.edu (R.J.H.); 3 Khoo Teck Puat-National University Children’s Medical Institute, National University Health System, 5 Lower Kent Ridge Road, Singapore 119074, Singapore; 4 Department of Pediatrics, University of California Davis School of Medicine, Sacramento, CA 95817, USA; 5 Department of Pathology and Laboratory Medicine, University of California Davis School of Medicine, Sacramento, CA 95817, USA; 6 Faculty of Medicine, University of Belgrade, 11129 Belgrade, Serbia; jelena.milisavljevic00@gmail.com (J.M.); fmitrovic0@gmail.com (F.M.); 7 Department of Psychiatry, Fragile X Clinic, Kennedy Krieger Institute, Baltimore, MD 21205, USA; 8 Department of Psychiatry & Behavioral Sciences-Child Psychiatry, Johns Hopkins School of Medicine, Baltimore, MD 21205, USA

**Keywords:** fragile X syndrome, *FMR1* gene, FMRP, behavior problems, autism spectrum disorder

## Abstract

Fragile X syndrome (FXS) is a neurodevelopmental disorder caused by the full mutation as well as highly localized methylation of the fragile X mental retardation 1 (*FMR1*) gene on the long arm of the X chromosome. Children with FXS are commonly co-diagnosed with Autism Spectrum Disorder, attention and learning problems, anxiety, aggressive behavior and sleep disorder, and early interventions have improved many behavior symptoms associated with FXS. In this review, we performed a literature search of original and review articles data of clinical trials and book chapters using MEDLINE (1990–2021) and ClinicalTrials.gov. While we have reviewed the biological importance of the fragile X mental retardation protein (FMRP), the FXS phenotype, and current diagnosis techniques, the emphasis of this review is on clinical interventions. Early non-pharmacological interventions in combination with pharmacotherapy and targeted treatments aiming to reverse dysregulated brain pathways are the mainstream of treatment in FXS. Overall, early diagnosis and interventions are fundamental to achieve optimal clinical outcomes in FXS.

## 1. *FMR1* Gene and Fragile X Mental Retardation Protein

The fragile X mental retardation 1 gene (*FMR1*; HUGO Gene Nomenclature Committee (HGNC) name: FMRP translational regulator 1) is located on chromosome Xq27.3. The normal range of CGG trinucleotide repeats in the *FMR1* gene is 5–44, and encodes the fragile X mental retardation protein (FMRP) [1,2,3]. FMRP has a variety of functions, many of which are critical for neurological development and function. Having a central role in brain development, FMRP binds to ribosomes and regulates translation of specific messenger RNAs (mRNAs) involved in neuronal synapse formation. Irwin and colleagues (2000) emphasized that FMRP serves as an ‘immediate early protein’ at the synapse that orchestrates aspects of synaptic development and plasticity. Additionally, FMRP regulates RNA stability and subcellular transport [4,5,6,7,8], and it also binds to multiple ion channels to regulate their activity [9].

Many studies have shown that FMRP inactivation interferes with dendritic spine formation via alteration of neural mRNAs, leading to disruption of neural protein synthesis. Inactive or altered forms of FMRP influence synaptic organization and connectivity, manifesting as deficits in quantity and integrity of neuronal dendrites and dendritic spines [7,10,11,12]. Greenough and colleagues (2001) described that dihydroxyphenylglycine-activated protein synthesis in synaptoneurosomes is reduced in animal model of fragile X syndrome (FXS) suggesting that FMRP is involved in or required for this synthesis [13]. FMRP inactivation may also lead to imbalance between neuronal excitation and inhibition. First, the loss of the FMRP in neural cells increases expression of glutamate receptors. FMRP synthesis is increased by activity of metabotropic glutamate receptor 5 (mGluR5), which in turn serves as a negative feedback mechanism to regulate mGluR activity [7,14]. Second, the absence of FMRP has been shown to decrease synthesis of both gamma-aminobutyric acid (GABA) and its receptor [15]. Therefore, an imbalance of these neurotransmitters may contribute to the disruption of neural plasticity [16]. FMRP has also been linked to ion channels regulation. Through interaction with the sodium-activated potassium Slack channels, FMRP plays an indirect role in neurotransmitter release via regulating action potentials through the large conductance Ca^2+^-activated potassium BK channel [5,17,18,19]. Additionally, the targets of FMRP are voltage-gated potassium channels Kv3.1b and Kv4.2 mRNA [19,20]. All functions discussed here are critical for efficient synaptic communication. In the absence of FMRP, weak synaptic connections are unable to undergo sufficient neural plasticity, thereby halting normal intellectual development. Finally, small amounts of FMRP were discovered in the cell nucleus, suggesting FMRP may have many more, previously unknown functions. This is supported by several studies suggesting FMRP’s role in regulating DNA expression via DNA stabilization and epigenetic regulation, through the protein’s effects on RNA regulation and cellular response to DNA damage [5,21].

The following sections describe pathophysiology and clinical presentation of FXS, as well as a variety of therapeutic approaches.

## 2. The Pathophysiology of Fragile X Syndrome

FXS is the most common form of inherited intellectual disability (ID) and monogenic cause of Autism Spectrum Disorder (ASD) [22]. The prevalence rate of FXS is estimated at 1 in 5000 males and 1 in 8000 females [6]. It is caused by the full mutation (FM) of the *FMR1* gene, which is characterized by the excessive expansion of CGG trinucleotide repeats (≥ 200) in the 5’ untranslated region (UTR) of the gene. These expanded CGG triplet repeats are hypermethylated with consequent transcriptional gene silencing, halting gene expression, thereby resulting in a reduction or absence of FMRP [23,24]. Although this is considered the main cause of FXS, many of the numerous molecular mechanisms involving FMRP and some physiological consequences presenting as FXS are yet to be discovered [5]. It is well known that mild to moderately low FMRP levels are associated with less severe symptoms, such as moderate emotional dysregulation and learning difficulties, often with a normal IQ, as is seen in some girls with FXS. Extremely low levels of FMRP or lack of its synthesis are associated with more severe forms of ID, as is common in males with FXS [25,26,27].

The number of CGG trinucleotide repeats expands with each subsequent generation, growing from premutation in women (PM; 55–200 repeats) to a FM in their offspring [28]. For PM alleles with more than 99 CGG repeats, the risk of transition from PM to FM approaches 100%. Individuals with PM have a normal IQ, while it is observed that female PM carriers have a high probability of having a child with FXS [24,29,30]. It has also been shown that neurons with PM undergo earlier cell death in culture, with heightened toxin sensitivity. For example, these neurons are more vulnerable to toxins in the environment, such as alcohol and pesticides, and they die more readily in cell culture [31,32,33]. “Gray zone” or intermediate alleles of the *FMR1* gene (45–54 CGG repeats) could be considered precursors for PM alleles [24]. The tendency of trinucleotide repeats to expand with each generation is why this genetic change is referred to as a ‘dynamic mutation’ [24].

There are multiple animal models used to study Fragile X: *Fmr1* knock-out (KO) rat and mice models, *Drosophila melanogaster* model of fragile X, and *Fmr1* KO zebrafish [34,35]. More than 20 years ago, the *Fmr1* KO mouse model was developed to help understand FXS. Research in the mouse model have revealed that the *Fmr1* gene is involved in plasticity and synaptic formation, which has led to the identification of potential drug targets for FXS. Long-term potentiation (LTP) and long-term depression (LTD) are forms of synaptic plasticity that are essential in learning and memory. Dahlhaus (2018) reviews numerous studies which found that LTP is impaired in the cortex of mouse models of FXS, and mGluR1/5-mediated LTD is enhanced in the hippocampus [34]. Deleting FMRP drives deficits in the N-methyl-D-aspartate receptor (NMDAR)-mediated synaptic plasticity in *Fmr1* KO mice [36]. 2-Methyl-6-(phenylethynyl) pyridine (MPEP), an mGluR-5 antagonist, has been shown to reverse spatial memory deficits in *Fmr1* KO mice and increase the synaptic marker PSD95. Importantly, stimulation of NMDAR and mGluR5 also hyperactivates the mTORC1 pathway, promoting the translation of matrix metalloproteinase 9 (MMP-9), which drives the degradation of extracellular proteins involved in synaptic function and maturation [37,38]. MMP-9, abundant in the brains of individuals with FXS as well as *Fmr1* KO mice, interferes with synaptic plasticity. Metformin, which lowers the abnormal elevation of the mTORC1 pathway observed in animal models and in individuals with FXS, can also reduce elevated MMP-9 levels. [37].

*Fmr1* KO zebrafish are also an ideal model often used to study ASD and social behaviors because they form groups called shoals. Studies in the zebrafish recapitulate the *Fmr1* studies on transgenic mice. For example, the zebrafish model shows a decrease in LTP and an increase in LTD [39]. Moreover, the behavioral characteristics such as anxiety and hyperactivity observed in individuals with FXS and ASD were also observed in the zebrafish model [40]. These transgenic fish display increased anxiety in novel fish tanks and changes in shoaling behavior due to hyperactivity [41]. In *Fmr1* KO zebrafish larvae, abnormal craniofacial development was also observed [42].

In the *Drosophila* fragile X model, the single ortholog of *FMR1*, *dfmr1*, has been mutated and causes the absence of dFMRP. This model has been extremely fruitful in the context of better understanding FXS pathogenesis. It displays several relevant phenotypes, including defects in the circadian output pathway, sleep problems, memory deficits in the conditioned courtship and olfactory conditioning paradigms, social interaction deficits (with peers and in naïve courtship), and deficits in neural development. Furthermore, signaling pathways found to be altered in the *dfmr1* mutant fly are often also altered in the mouse FXS model [43,44,45,46].

### Brain Structure and Neuronal Morphology

Brain MRI of individuals with FXS have demonstrated larger brain volumes with larger lateral ventricles compared to those without FXS. One of the most apparent features is cerebellar vermis hypoplasia, meaning the vermis is smaller in size [47]. Measurements of apical dendritic spines of layer V pyramidal neurons in temporal cortex of human autopsy samples of male patients with FXS showed that they were significantly longer overall and exhibited a morphology consistent with that of early development: a greater number of long spines with heads and fewer short, stubby, and mushroom-shaped spines [13,48]. In addition, nonquantitative observations of rapid Golgi-stained human autopsy material from a single patient with FXS described long, thin, tortuous, dendritic spines with prominent heads and irregular dilations on apical dendrites of pyramidal cells in layers III and V of parieto-occipital cerebral cortex. Reduced mean synaptic contact area was also reported, based on electron microscopic observations [13,49,50].

The described pathophysiology and changes in brain structure and neuronal morphology lead to the clinical phenotype found in patients with FXS as presented in the following section.

## 3. Clinical Presentation of Fragile X Syndrome

While some parents notice developmental delays during the child’s first year of life, they are more obvious in the second or third years of life particularly with language delays. The mean age of FXS diagnosis is 36 months in the United States [5,51].

**Figure 1:**
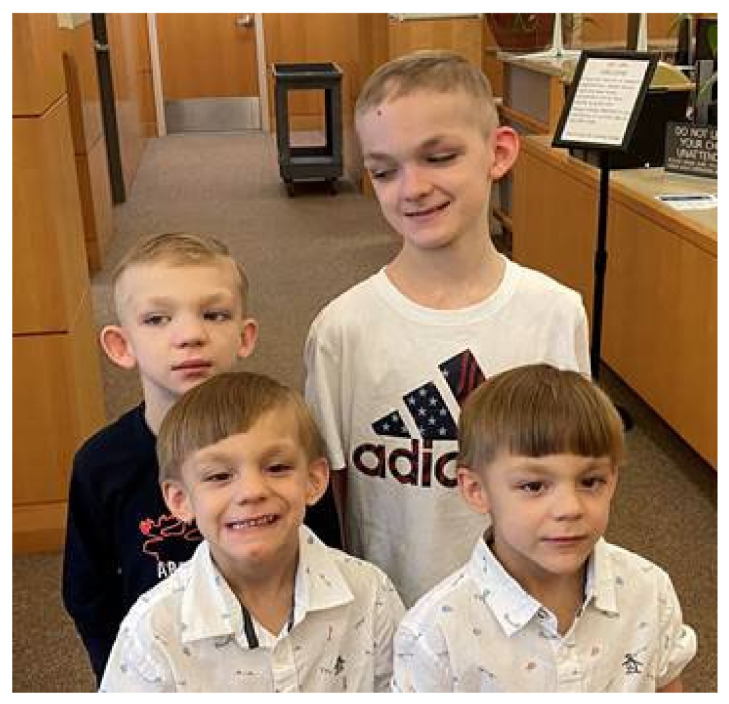
Four brothers with typical physical features of FXS. All four boys have prominent broad foreheads, high palate, joint hypermobility and especially prominent ear pinnae. The family gave consent to publish this picture.

The clinical presentation of FXS differs by gender. Males are generally more affected, while females tend to present with a less severe phenotype due to compensatory activation of the unaffected X chromosome [25]. Consequently, only 25 to 30% of females with FXS are affected by ID, although another 30% have a borderline IQ and those with a normal IQ often have emotional problems or learning disabilities [25]. In general, FXS manifests as a variety of neurobehavioral conditions. These manifestations may be further divided into physical, psychological, or classified as a form of ASD. Over 80% of individuals with FXS have characteristic physical features, including an elongated face and large or prominent ears (see Figure 1), high palate, joint hypermobility and macroorchidism at puberty or thereafter [47]. The most important FXS manifestations are: ID, speech and language delay, ASD, social anxiety, shyness, abnormal eye contact, sensory hyperarousal, attention deficit hyperactivity disorder (ADHD), aggressive behavior, sleep problems, seizures, hand flapping, repetitive behaviors or even obsessive–compulsive disorder (OCD). More than 90% of boys with FXS suffer from developmental delay and 50–60% are diagnosed with ASD [22,52,53,54]. About 15 to 20% of individuals with FXS have seizures, especially those with ASD [55]. In addition, over 30% of patients suffer from obesity and gastrointestinal dysfunction such as gastroesophageal reflux [33].

Physical features of FXS are usually not apparent at birth as neonates with FXS often have no clinical signs except for hypotonia, which is common [56]. During early childhood, physical and developmental features of FXS become more apparent. Delays in motor, speech and language development and autistic features are typical by 3- to 4-year-old, and should lead to diagnosis [57]. Nevertheless, health professionals are often unfamiliar with these features and FXS [58,59,60].

There is a close correlation between the presence of FXS, ASD and ADHD [47,61,62]. Over 60% of boys with FXS are co-diagnosed with ADHD, ASD or both. Based on reports from previous studies, approximately 40–67% of males and 20–23% of females with FXS are diagnosed with ASD [53,63,64]. Furthermore, among those individuals with FXS who do not meet criteria for the diagnosis of ASD, up to 90% may exhibit some form of behavioral symptoms associated with ASD, such as hand flapping or hand biting or poor eye contact [25]. While FXS is the most common monogenic cause of ASD, only 2–6% of all ASD cases are caused by FXS [64].

Boys with FXS and comorbid ASD present with more severe forms of developmental delay and behavioral difficulty [25]. Those boys with FXS and ASD are more frequently reported to suffer from ADHD and anxiety, compared to boys with ASD without FXS [25]. There are also differences between the presentation of ASD related to FXS, compared to that of ASD without FXS. For example, McDuffie et al. found that boys with FXS show less impairment in social smiling and gesture use, while boys with ASD without FXS show less impairment in complex mannerisms [64]. Additionally, clinical trials demonstrate that individuals affected by FXS and ASD do not respond equally to the same treatment, suggesting different molecular mechanisms may be responsible for shared symptomatology [65,66], even though overlapping neurobiological pathways are known to be affecting individuals with FXS and idiopathic ASD [25]. The overlap of molecular mechanisms in those two groups suggests an interaction between various signaling pathways during brain development. Known shared neurobiological pathways currently being studied to target specific pharmacological treatments for FXS and ASD include (1) the Wingless (Wnt) signaling pathway and pathway regulators involved in processes of neurogenesis and cell signaling. Abnormalities in this pathway lead to ASD-like behavior, including anxiety, cognitive impairment, difficult social interactions, and repetitive behaviors. Many of the commonly used medications to treat challenging behaviors in FXS and ASD modulate this pathway, including SSRIs, antipsychotics and stimulants, (2) The PI3K/AKT/mTOR (phosphoinositide 3-kinase/protein kinase B/mechanistic target of rapamycin) pathway. In the brain, mTOR is thought to be involved in the development of neuronal circuitry, synaptic plasticity, and regulation of complex behaviors (3) ERK/MAPK pathway. Medications like lovastatin and metformin modulate the PI3K/AKT/mTOR and ERK/MAPK pathways. (4) The endocannabinoid system has also been found to be dysregulated in ASD and FXS. Pharmacological modulation of this pathway shows improvement in symptoms of anxiety, poor sleep and seizures [67].

Genetic diagnosis of FXS relies primarily on molecular techniques. PCR and Southern Blotting are frequently used to detect the presence of *FMR1* gene mutations in individuals with suspected FXS. PCR plus Southern blotting are considered the ‘gold standard’ for diagnosing FXS, and is most commonly used for detection of CGG triplet expansions. The PCR technology involves using specific primers complementary to the expanded CGG sequences, allowing amplification and subsequent detection. With newer PCR techniques it is possible to identify both individuals with PM and FM using PCR [26,68]. These analyses may be further applied to assess characteristics of alleles in the FM range, and to discover the presence and patterns of methylation [5,24,69].

Fragile X DNA testing detects more than 99% of individuals with disorders associated with FXS [28] and a rare patient may have a point mutation in *FMR1* that may only be seen by whole genome sequencing or whole exome sequencing. The National Fragile X Foundation (NFXF) offers helpful guidelines on fragile X testing, available at https://fragilex.org/fragile-x/testing/ (accessed on 18 December 2021). As per this guideline, in addition to specific indications for testing, there are three general circumstances in which Fragile X testing should be considered: (a) clinical symptoms suggestive of FXS or another disorder associated with fragile X including premutation conditions; (b) family history of FXS, intellectual or learning disabilities, or autism of unknown cause; and (c) family or personal history of a Fragile X mutation in the extended family. Similar guidelines for fragile X testing come from The American College of Medical Genetics and Genomics (ACMG) [70], Consensus guidelines from the American Academy of Pediatrics (AAP) from 2011, EMQN best practice guidelines [71], AACAP [72] and others.

Regardless, after receiving the diagnosis of developmental delay due to FXS, early non-pharmacological interventions in combination with pharmacotherapy, when necessary, are crucial for most children with FXS.

## 4. Non-Pharmacological Therapy of FXS

Non-pharmacological early interventions for FXS such as physical therapy (PT), occupational therapy (OT) and speech-language therapy (SLT) therapies are critical in addressing gross and fine motor and speech-language delays, respectively, as well as Applied Behavior Analysis (ABA) for social-communication deficits in co-morbid ASD [53]. In children and adolescents, behavioral (parent training) interventions are valuable in reducing behavioral problems such as aggression, hyperactivity, and tantrums. In females and higher functioning males’ individual therapy, including cognitive behavior therapy (CBT) can help anxiety, ADHD, social deficits and depression [53]. The benefits of these non-pharmacological therapies can be recognized in children with FXS with or without ASD, although those with ASD are more likely to benefit from intensive ABA therapy. These programs are often started in the preschool years through a special education program or in an early start home program [47]. The interventions described below are some non-pharmacological therapies available that have been used to assist in educational development and improvements in adaptive behaviors.

SLT and OT are the two most common services used by children with FXS [53,73]. A large portion of children with FXS utilize SLT to address the delay in communication development. Speech therapy games have shown to enhance the control of one’s pitch [74]. Furthermore, parents and teachers observed a marked elevation of social communication in children with ASD following speech therapy [75].

OT for individuals with FXS focuses on improving the sensory integration problems that are seen in those with FXS. In young children, therapists work on enhancing sensory processing, sensorimotor abilities, and participation in play. OT with a sensory integration approach addresses the sensory hyper-reactivity observed in children with FXS [47]. In adolescents, independence and vocational skills are emphasized [76]. Studies have found that vocational programs that teach life skills such as community participation, computer skills, money management, and social interaction led to long-term benefits of individual satisfaction and stable employment [74,77,78].

Parental Implemented Language Intervention (PILI) is a parent-implemented, narrative-based language intervention developed to address deficits in the use of pragmatically appropriate language and limitations in understanding inferential language in social interactions common to children with FXS with and without co-morbid ASD [79,80]. This novel language intervention uses a shared storytelling approach and has demonstrated to be beneficial to increase prompted inferential language in school-aged children with FXS as well as to support parental acquisition of strategies that can mediate the effect of the language intervention on their children [81].

ABA is another intervention that is used for children with FXS, especially those with ASD. The theory behind ABA is the reinforcement of favorable behaviors [82]. The Early Start Denver Model (ESDM) is a behavioral therapy, based on ABA principles, that enhances social interactions and language on a daily basis in a naturalistic setting at home and targeted for young children between 12 and 48 months. Numerous studies have found that ESDM improved language, cognitive function, adaptive behavior, and social communication in children with ASD [83]. A pilot study on a parent-delivered ESDM in children with FXS reveals gains in fine motor skills, verbal expressivity, visual reception, and adaptive behavior. Furthermore, the parents reported a better understanding of their children’s way of thought and communication [83,84].

PT is the third most frequent intervention used by children with FXS [73]. Special treatment considerations for children with FXS are often given, which require intermittent assessment of motor development as well as hypotonia and joint hypermobility [85]. To address delays in walking skills and balance, physical therapists may recommend assistive devices such as orthotics to correct foot pronation or strollers for stability [85].

Exercise has been shown to be beneficial for children with FXS and ASD [86]. In neurodevelopmental children, physical activity is associated with improved attention and cognitive control [87]. This may be explained by the association between exercise and an increase in brain-derived neurotrophic factor (BDNF), which is essential in brain development and neuroplasticity [88]. Research in mice models and in vitro experiments reveal a synergistic interaction between BDNF and *FMR1*. BDNF induces *FMR1* transcription, and the absence of FMRP can alter BDNF signaling and delay brain development [89]. Increased BDNF through exercise therefore aids in brain development and improves cognitive control.

CBT is a form of talk therapy that has been effective in emotional regulation and can be used to address the high levels of anxiety in children with FXS. Current models that describe its mechanism postulate that CBT treats anxiety through fear extinction, habituation, and inhibitory learning [90]. Studies have shown that CBT improves emotional regulation and symptoms of anxiety and depression for children with ASD [91].

Music therapy has also been used by children with FXS to develop communication skills and improve self-expression [92]. It has shown to increase verbal communication, social interactions, and social–emotional reciprocity in people with ASD [93]. Listening to and creating music may help non-verbal children with FXS express themselves through a unique avenue that encourages creative expression of emotions [93].

**Figure 2:**
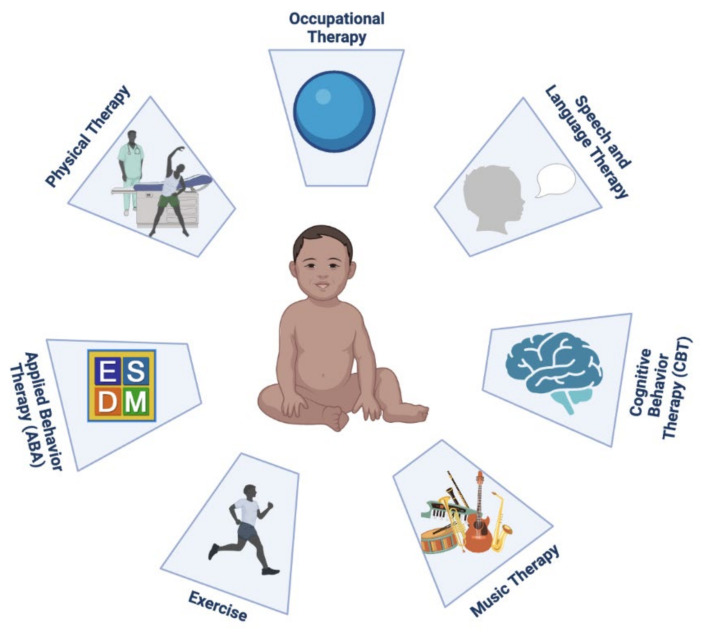
Schematic presentation of non-pharmacological interventions for fragile X syndrome. (Created with BioRender.com, accessed on 18 December 2021).

Figure 2 is a schematic presentation of non-pharmacological interventions for FXS.

## 5. Psychopharmacology in FXS

The most common psychiatric symptoms that are sought to improve with drug treatment in FXS are: ADHD, anxiety, aggressiveness and sleep problems. In general, individuals with FXS may have greater side effects to medications at a given dose compared to the general population, hence starting any pharmacological agent at a low dose and gradual titration upwards as needed for symptom reduction is recommended [94,95].

### 5.1. Attention Deficit Hyperactivity Disorder (ADHD) Symptoms

Attention Deficit Hyperactivity Disorder (ADHD) is characterized by a persistent pattern of inattention and/or hyperactivity and impulsivity that are more inappropriate or disruptive than those in other children of a comparable age resulting in functional impairment in multiple settings [96].

Stimulants are the first-choice agents to address ADHD symptoms in children older than 6 years of age. Stimulants exert their action by increasing levels of dopamine and norepinephrine in the pre-frontal cortex through effects at the neuro-transmitter level [97]. Both these neurotransmitters are beneficial for improving attention, increasing task motivation and improving impulse control [98]. Stimulants are effective in ~70% of individuals with ADHD symptoms and are effective at similar doses as in the general population in individuals with FXS [99]. The main classes of stimulants include methylphenidate or amphetamine derivatives and are usually well tolerated in FXS, especially in children older than 5 years [100]. Common side effects are generally minor with change in appetite and gastro-intestinal discomfort being the most common [101]. Serious but rare adverse effects include palpitations and raised blood pressure; however, these are rare, with an incidence rate for serious cardiovascular effects of 3.1 per 100,000 person years in one large population-based study [102]. Of note, several studies demonstrated no added risk of sudden cardiac-related death due to stimulants in the FXS population compared to the general population [103,104].

Alpha 2-adrenergic receptor agonists, namely clonidine and guanfacine, can also be useful for ADHD symptoms [105]. These are particularly useful in individuals who do not respond to or are not able to tolerate stimulants such as children younger than 5 years of age. These agents act at the alpha 2-adrenergic receptor agonists in the pre-frontal cortex to increase norepinephrine levels; this in turn has beneficial effects on attention modulation and in FXS they have a calming effect for hyperarousal [106]. Clonidine can be helpful for children with FXS who also have sleep disturbances, and sleep problems [99]. Guanfacine, has less sedation compared to clonidine so it is used more often to calm behavior during the day than clonidine [99]. Possible side effects for both drugs include sedation and hypotension after treatment initiation; hence, abrupt withdrawal should be avoided to minimize the risk of rebound hypertension [99].

### 5.2. Anxiety Symptoms

Anxiety disorders are common in individuals with FXS, with 70 to 80% having some form of anxiety [107]. Selective serotonin reuptake inhibitors (SSRIs), like sertraline are very effective for this purpose [65,99]. These exert an inhibitory action at the presynaptic serotonin reuptake terminals resulting in an increase in serotonin levels in the synapse, which is a key positive mood regulator within the central nervous system [108]. Sertraline can be started for anxiety at a young age and has been found to have additional beneficial effects for language and motor development and receptiveness to other therapies in addition to anxiety [65,109,110]. Sertraline is generally well-tolerated, but common side effects include GI symptoms (i.e., stomachache, diarrhea, loss of appetite). In children, symptoms of activation with agitation and restlessness can occur occasionally, especially with rapid titration of dosage. Start-low-go-slow approach typically minimizes such risk, and reduction in dose and/or discontinuation of the drug may be indicated if this occurs and persists.

Bupropion is another anti-depressant that increases the level of both noradrenergic and dopaminergic neurotransmission via re-uptake inhibition [111]. Bupropion can address symptoms of depression and anxiety especially in individuals concerned with weight gain with SSRIs; placebo-controlled trials of individuals with depression have shown that bupropion helps with weight loss as well [112,113]. Bupropion can also help with symptoms of ADHD including inattention, but should not be given in those with seizures or a propensity for seizures. Possible side effects include xerostomia, restlessness and insomnia.

### 5.3. Aggressive Behavior

Aggression, self-injurious behaviors and explosive temper tantrums outbursts (“meltdowns”) can be seen in individuals with FXS, especially in the teenage years [114]. Atypical antipsychotics including risperidone and aripiprazole can help to address these symptoms of irritability accompanied by aggression, and the meltdowns. They exert their effects through neural dopaminergic and serotonergic receptors, although their exact mechanism of action is not fully understood [99,105]. Both these agents are generally safe and well tolerated with weight gain being the main side effect; aripiprazole has a lower propensity for weight gain and may thus be favored over risperidone [115]. This is an important fact to account for when treating individuals with FXS, since obesity is found in 30–60% of individuals with FXS and often compulsive eating behaviors which emerge as a coping mechanism for anxiety and irritability also contribute to weight gain [116,117]. Future molecular studies ought to compare a head-to-head differential effects of antipsychotics [118].

### 5.4. Sleep Problems

Sleep problems are common in individuals with FXS. Between 27 and 77% of individuals with FXS suffer with some form of sleep difficulty [119,120]. Melatonin is the primary drug of choice for sleep problems. Melatonin is an endogenous neurohormone mainly synthesized and released by the pineal gland at night under normal conditions [121,122], its primary physiological function involves regulating the circadian rhythm and its sleep-promoting actions are mostly caused by its feedback to the melatonin receptors in the suprachiasmatic nucleus [123]. Melatonin can help to shorten sleep-onset latency and increase longer night sleep duration. Melatonin also has anti-oxidative properties and can facilitate synaptic plasticity and these can potentially help with learning and memory [124]. Melatonin is usually well-tolerated with minimal side effects; but day-time drowsiness and nausea have been reported in some individuals.

Table 1 presents a summary of the commonly used medications in FXS, their mechanism of action, dosage guides and side effects [95].

### 5.5. Seizures

In general, seizures are well-controlled with a single anticonvulsant in individuals with FXS. Treatment involves the use of a range of anticonvulsant medications. However, levetiracetam and oxcabazepine are commonly used as first-line treatment due to their good side effect profiles. Other medications such as lamotrigine and valproic acid could be useful particularly in those with generalized seizures. On the other hand, phenytoin, phenobarbital and gabapentin should be avoided because of their adverse effects’ profiles. Levetiracetam occasionally worsens irritability and aggressive behavior, although some individuals with FXS have positive behavioral responses in addition to an anticonvulsant response. Individuals with FXS who are experiencing significant adverse effects should be switched to other anticonvulsants due to availability of a wide range of these medications with good safety profiles [55,94,99].

## 6. Targeted Treatments

The animal models have guided the use of targeted treatments that can reverse the neurobiological abnormalities caused by the lack of FMRP in FXS. Excitatory (‘mGluR’ excess)/Inhibitory (‘GABA-ergic’ deficit) has been proposed and extensively studied in preclinical studies of FXS and subsequent multiple clinical trials [125,126,127]. Preclinical studies in *Fmr1* KO mice suggested that GABA receptor modulation may hold therapeutic potential for treating core problems associated with FXS such as altered synapse architecture, hyperactivity, hypersensitivity to auditory stimuli and increased seizure incidence [12,125,128]. For example, findings of decreased GABA type A (GABAA) receptor δ subunit expression in *Fmr1* KO mice were reversed using gaboxadol as a δ-subunit–selective extrasynaptic GABA_A_ receptor agonist [129]; recently, a signal-finding first interventional controlled clinical trial of gaboxadol in adolescents and adult males with FXS was published [130].

Another treatment target is group 1 metabotropic glutamate receptors (mGluRs), but several trials of two mGluR5-targeting therapies in adults with FXS, mavoglurant (noncompetitive mGluR5 inhibitor) and basimglurant (mGluR5 negative allosteric modulator, NAM), failed to demonstrate statistically significant differences from placebo on primary caregiver-rated behavioral efficacy outcomes [125,127]. Specifically, mavoglurant was tested in two phase II double-blind, placebo-controlled, parallel-group studies that included 175 adults aged 18–45 years and 139 adolescents with FXS. In both trials, participants were stratified by methylation status and randomly assigned to receive mavoglurant (25, 50 or 100 mg twice daily) or placebo over 12 weeks [125,131]. Basimglurant was tested in two phase II clinical trials in adult and adolescent patients aged 14–50 years and in children aged 5–13 years [125,132]. Both studies were designed as randomized, double-blind, placebo-controlled, parallel-design trials testing two doses of basimglurant over a 12-week treatment period in male and female patients. Questions remain as to whether these trials were conducted with the optimal outcome endpoints or in the most appropriate age group. Indeed, a follow-up assessment of these studies concluded that future research should (1) assess FXS for a longer duration (i.e., ideally 6 months) and in younger preschool age patients, (2) improve psychometric properties and sensitivity to treatment change(s) of existing and assess a broader range of preferably clinician-based outcome measures, and (3) develop new paradigms to quantify learning and try alternative study designs, (4) reduce phenotypic heterogeneity by applying patient stratification paradigms (i.e., FMRP level) and (5) try to better control for placebo effects and use biomarkers [27,125,127,133,134]. In the meantime, there is a progress in an effort to quantify mGluR5 expression in the living human brain of men with FXS to help understand results of those failed mGluR5 trials in humans with FXS, and to help provide information for successful clinical trial designs [135,136,137].

As no medication is approved by the US regulatory agency for the treatment of FXS, there remains a great need for safe and effective treatments for FXS, particularly for targeted treatments that surpass the symptom-based management [138].

### 6.1. Phosphodiesterase-4D (PDE4D) Inhibitor BPN14770

In the summer of 2021, an exciting paper was published [139] that demonstrated an improvement in cognition in 30 adult males (18–45 years old) with FXS who underwent a controlled trial of the PDE4D inhibitor (BPN14770) at a dose of 25 mg twice a day (NCT03569631). It has been known for many years that cAMP levels are low in those with FXS and this molecule inhibits the PDE4D enzyme that breaks down cAMP so that the levels of cAMP increased with the use of this medication.

This study used a novel outcome measure, the NIH toolbox, that has been modified for use with individuals with ID including those with FXS. Treatment with this PDE4D inhibitor for 14 weeks led to improvements in the Oral Reading Recognition, Picture Vocabulary and Cognition Crystallized composite scores from the NIH toolbox. The caregiver’s Visual Analogue Scale demonstrated significant improvements in language and daily functioning also. The primary outcome was the assessment of safety and this was a well-tolerated and safe medication without significant adverse effects compared to placebo. This is the first study that has shown cognitive improvements in adults with FXS so the field is energized for more studies of this compound. Currently multicenter phase 3 controlled trials are being carried out in children with FXS (NCT05163808) and later in 2022 further adult studies will be carried out.

### 6.2. Cannabidiol (CBD)

CBD is the non-psychotropic component in marijuana and it is available in marijuana stores and through the internet. It has been used widely by families to control anxiety, sleep disturbances and tantrums in their children with FXS or with ASD with anecdotal reports of benefit [140]. CBD has multiple effects in the endocannabinoid system including influencing the levels of the natural ligands, anandamide (AEA) and 2-arachidonoylglycerol (2-AG), for the cannabinoid receptors (CB1 and CB2). In the absence of FMRP the endocannabinoid system becomes dysregulated and is unable to maintain the balance of inhibitory and excitatory neurotransmitters, which is done through post synaptic release of AEA and 2-AG to stimulate the CB1 presynaptic receptors that occur throughout the CNS. CBD can increase 2-AG availability to signal the CB1 receptor, prevents internalization of the CB1 receptors and restores membrane expression of these receptors, increases AEA levels by reducing its access to the catabolic pathway, binds 5HT1A receptor with agonist efficacy, is a dopamine partial agonist and is a positive allosteric modulator of GABAA receptors. All of these effects help to restore the balance in excitatory and inhibitory pathways and improve behavior in FXS [141].

A CBD topical gel that does not have THC, the psychotropic component in marijuana, has undergone trials in FXS. The first trial of the topical gel, that has 125 mg of CBD per packet, which is rubbed on the shoulders twice a day, was an open label 12-week study in 20 children with FXS [142]. This phase 2 open label study demonstrated significant improvements in multiple behaviors including hyperactivity, aggression, anxiety and tantrums. These results stimulated a phase 3 controlled trial at multiple international centers in children ages 3 to 18 years old with FXS. The dose of CBD was 250 mg to 500 mg twice a day based on weight lasting for 12 weeks. Although the primary outcome measure of Social Avoidance (SA) on the Aberrant Behavior Checklist (ABC_FX_) was not met by the overall group of 212 subjects, a preplanned analysis in those with > 90% methylation had not only statistically significant improvement on the SA subtest, but also on the Caregiver Global Impression-Improvement (CGI-I) in multiple behaviors. This subgroup represented 80% of the study population and were the most affected of those with FXS, but it also included females who improved in addition to the males [141]. To gain FDA approval, however, another study (RECONNECT) (NCT04977986) is being carried out at multiple centers to confirm these results and those with >90% methylation will be involved in the primary analysis. These results demonstrate that CBD can be helpful for the majority of patients with FXS, particularly those that are fully methylated [143].

### 6.3. Metformin

A biguanide antihyperglycemic medication, metformin, is used in the treatment of type 2 diabetes mellitus (DM) and weight loss [144]. It is a first-line treatment for DM type 2. Metformin’s mechanism of action involves AMP-activated protein kinase (AMPK)-dependent and AMPK-independent pathways [144,145]. Moreover, metformin can modify the upregulated mGluR/mTORC1-ERK cascade in animal models of FXS. An improvement in social and cognitive behavior in these models involved the down-regulation of this mTOR pathway. Metformin also improved dendritic spine dysgenesis and morphological features, such as macroorchidism and electrophysiological abnormalities (long-term depression) [146,147]. In the study on Drosophila melanogaster FX model, metformin corrected circadian and cognitive deficits, improved through reduction of insulin signaling [46]. Metformin strongly inhibited mTOR through AMPK and rescued core deficits of the phenotype in the *Fmr1* KO mouse model of FXS [148].

Clinical data showed that metformin was well tolerated in patients with FXS that were treated clinically for at least 6 months. These patients had positive behavioral changes in areas such as irritability, social avoidance and aggression, as reported by caregivers; in addition to benefits in appetite and weight control in overweight patients [149]. Furthermore, nine children with FXS between 2 and 7 years of age who were treated clinically with metformin and monitored for behavioral improvements and metabolic changes also improved. Parent reports and developmental testing before and after metformin showed that there were improvements in language development and behavior (such as lethargy and stereotypy) in most of the patients. This report revealed the need for a controlled trial of metformin in children with FXS under 7 years old whose brains are in a critical developmental window and thus may experience a greater degree of clinical benefit from metformin compared to older patients [150]. Metformin also improved verbal and nonverbal IQ scores, after one year treatment in two adult males with FXS [151]. As mentioned above, metformin blocked the development of macroorchidism in the mouse model of FXS. Similar absence of macroorchidism was described after 2 years of metformin in an adolescent at age 14, in accordance with data obtained from the animal model [152].

A triplex protein array comprising of hexokinase 1 (HK1), RAS (all isoforms), and Matrix Metalloproteinase 9 (MMP9) were tested to assess the ability of molecular biomarkers to predict phenotypic subgroups, symptom severity, and treatment response to metformin in clinically treated patients with FXS. This study included 17 participants with FXS (12 males and 5 females) treated clinically with metformin. The disruption in expression abundance of these proteins was normalized and associated with significant self-reported improvement in clinical phenotypes (CGI-I in addition to body mass index) in a subset of participants with FXS. These preliminary findings suggest that these proteins are of strong molecular relevance to the FXS pathology that could make them useful molecular biomarkers for FXS and targeted treatments [153].

The results obtained in an open-label study which included 15 patients with FXS aged from 17 to 44 who received 500 mg of metformin twice/daily over a 9-week treatment period showed that metformin treatment was well tolerated, with no significant related side effects. The Transcranial Magnetic Stimulation parameters showed an increase in corticospinal inhibition mediated by GABA_A_ and GABA_B_ mechanisms. This study demonstrated the safety of metformin in normoglycemic patients with FXS, and suggested the potential of this medication in modifying GABA-mediated inhibition, a hallmark of FXS pathophysiology [154].

Results from the first double-blind randomized controlled trial, being conducted in United States and Canada (NCT03862950, NCT03479476), evaluating the safety along with the efficacy of metformin in the treatment of language deficits and behavior problems in individuals between 6 and 40 years of age with FXS will be available by 2022. Metformin appears to be a strong candidate for a new targeted treatment for FXS.

### 6.4. Minocycline

Minocycline is a second-generation, semisynthetic tetracycline derivative with long half-life and lipophilic characteristics that allow it to cross the blood–brain barrier [155,156]. In addition to its antibiotic actions, minocycline also has central anti-inflammatory properties via inhibition of several molecules, including COX-2, iNOS, and p38 MAPK, and antiapoptotic functions via caspase inhibition, among other mechanisms of action [155], conferring neuroprotective properties.

Early preclinical studies in Drosophila FXS model with minocycline showed its effectiveness in restoring normal synaptic structure in the neuromuscular junction, clock neurons in the circadian activity circuit and Kenyon cells in the mushroom body learning and memory center [157]. In addition, minocycline showed reversal of ultrasonic vocalizations during mating, suggestive of abnormal social communication in Fmr1 KO mice through attenuation of MMP-9 levels [158]. A randomized crossover trial conducted in a pediatric group with FXS (NCT01053156), ages 3.5–16 years demonstrated improvements in global functioning measured by CGI-I. Anxiety and other mood-related behaviors were perceived to be improved by parents [159] in addition to changes in event-related potentials (ERP) where electrocortical habituation to auditory stimuli demonstrated improvement after at least 3 months of treatment with minocycline [160]. Common side effects reported during the trial include diarrhea, skin rash, GI upset, and blue-grey, brown and yellow tooth discolorations [159].

Table 2 presents a summary of new drugs under clinical trials in fragile X syndrome.

## 7. Conclusions

Early non-drug interventions in combination with symptom-based and core-symptoms targeted treatments pharmacotherapy is the main management in individuals with FXS. The latter aims to reverse underlying dysregulated brain pathways found to be the cause of problem behaviors and cognitive impairments in FXS. In this review, we compiled both symptom-based and targeted pharmacological interventions that have proven to be beneficial in the management of common psychiatric symptoms including ADHD, anxiety and aggression found in FXS. In addition, we emphasize that early non-pharmacological interventions are still primary to achieve optimal outcomes in a variety of skills and behaviors. Use of targeted treatments in FXS has shown benefits in multiple problem behaviors found in the FXS behavioral phenotype. While some of those targeted available drug compounds have revealed improvements in overall clinical impression, further improved designs of clinical trials to include objective outcomes and biomarkers paired with learning paradigms promise to further elevate the effort toward serving unmet needs of individuals with FXS.

## Figures and Tables

**Table 1:** Summary of common pharmacological agents used in fragile X syndrome against the most common psychiatric symptoms (out from [95]).

Medication	Drug Class	Mechanism of Action	Target Problems	Dose/Day	Common Side Effects
*Sertraline*	Selective serotonin reuptake inhibitor (SSRI)	Inhibition of presynaptic neuronal uptake of serotonin (5HT)	Anxiety, aggressive behaviors, social participation, language development in young children	2.5 to 5.0 mg in young children (2 to 6 years) 10 to 100 mg in older children and adolescents, up to 200 mg per day in adults	Diarrhea, loss of appetite, hyperhidrosis, activation (restlessness, mood changes, disinhibited behaviors), tremor
*Methylphenidate*	Central nervous system (CNS) stimulant	Non-competitively blocks the reuptake of dopamine and noradrenaline	Attention deficit and hyperactivity disorder (ADHD)	10 to 60 mg per day typically depending on the preparation and dosing frequency	Decreased appetite, nausea, headaches, hypertension, irritability, insomnia
*Clonidine*	Alpha 2-adrenergic receptor agonist	Stimulates presynaptic and postsynaptic alpha 2 adrenergic receptors in the prefrontal cortex	Hyperactivity, overstimulation, attention/concentration problems and aggression, sleep disturbances	Initial dose 0.025 mg/day in children and titrate to maximum dose of 0.4 mg/day in older children and adults	Sedation, postural hypotension, nausea, constipation, bradycardia, xerostomia
*Guanfacine*	Alpha 2-adrenergic receptor agonist	Stimulates presynaptic and postsynaptic alpha 2 adrenergic receptors in the prefrontal cortex	Hyperactivity, frustration intolerance, hyperarousal	Initial dose of 0.5 mg/day and titrate up to a maximum dose of 4 mg/day in adults	Less sedation than clonidine, nausea, constipation, bradycardia, xerostomia
*Risperidone*	Second-generation antipsychotic	Blocks dopamine D2 receptors in the prefrontal cortex and nucleus accumbens. Serotonin and norepinephrine reuptake inhibition	Irritability, aggression, self-injury, social impairment, stereotypic behaviors, psychosis and hyperactivity	0.5 to 3 mg/day based on weight. Available in long-acting injection	Weight changes, metabolic changes, and sedation. Extrapyramidal symptoms, parkinsonian features, hyperprolactinemia
*Aripiprazole*	Second-generation antipsychotic	Blocks dopamine D2, D3 and 5-HT1A (serotonin) receptors. Antagonist at the 5-HT2A receptor	Irritability, aggression, agitation, and self-injurious behaviors, sleep problems, severe anxiety	2 to 15 mg/day based on weight and age	Increase in weight, somnolence (dose–response relationship) and extrapyramidal symptoms
*Melatonin*	Biogenic amine/endogenous hormone	Activates melatonin receptors ML1/ML2, leading to inhibition of adenylate cyclase and the cAMP signal transduction pathway; Activation of phospholipase C	Sleep disturbances	Initial low dose of 1 mg in young children and titrating up as needed; maximum dose 10 mg typically	Day-time drowsiness, headache, dizziness, nausea

**Table 2:** Summary of new drugs under clinical trials in fragile X syndrome [95].

Medication	Drug Class	Mechanism of Action	Target Problems	Dose/Day	Common Side Effects
*Cannabidiol (CBD)*	Phytocannabinoid exhibits no effects indicative of any abuse or dependence potential	Multiple: Interact with an FXS-compromised endocannabinoid system; positively affect synaptic plasticity; a positive allosteric modulator of GABAA receptors; Serotonin 1A receptors modulator	Multiple: Reductions in social avoidance and anxiety; improvements in sleep, feeding, motor coordination, language skills, anxiety, seizures and sensory processing	Oral tincture: 25 to 50 mg per dose up to bid Topical ointments: 125 mg to 500 mg bid	Sedation, but rare cases of activation. Higher doses may cause liver function test elevations. Topicals may cause skin rash or irritation
*BPN14770*	*Phosphodiesterase-4D (PDE4D) inhibitor*	Selectively binding to and partially suppressing the activity of phosphodiesterase 4D (PDE4D), an enzyme known to regulate a brain cyclic adenosine monophosphate (cAMP).	Cognitive function, language and daily functioning	Adult: 25 mg bid	The most commonly reported side effects were vomiting and upper respiratory infections
*Metformin*	Oral antidiabetic; Class: biguanides	Impacts mTOR and ERK/MAPK pathways through the AMP- activated protein kinase pathway. Decreases the level of protein MMP-9 in FXS	Intellectual abilities, behavioral problems, speech and language deficits, weight management	500 mg bid < 50 kg 1000 mg bid > 50 kg	Gastrointestinal side effects (nausea, diarrhea and abdominal discomfort), headache, weight loss, dizziness, inadequate vitamin B12
*Minocycline*	Antibiotic; Class: tetracycline	Anti-inflammatory, MMP inhibition and anti-apoptotic effects. Inhibition of activity of the protein MMP-9 in FXS	Overall clinical improvement; In particular, mood and anxiety-related behaviors	25 mg < 25 kg 50 mg 25–45 kg 100 mg > 45 mg	Gastrointestinal side effects (nausea, diarrhea, etc.), dizziness, discoloration of skin, nails or gums

## Data Availability

Not applicable.
